# Prevalence and predictors of problematic alcohol use, risky sexual practices and other negative consequences associated with alcohol use among safety and security employees in the Western Cape, South Africa

**DOI:** 10.1186/1747-597X-9-14

**Published:** 2014-03-04

**Authors:** Nadine Harker Burnhams, Charles Parry, Ria Laubscher, Leslie London

**Affiliations:** 1 Alcohol and Drug Abuse Research Unit, South African Medical Research Council, P.O. Box 19070, Cape Town 7505, South Africa; 2 Department of Psychiatry, Stellenbosch University, Tygerberg, PO Box 19063, Cape Town 7505, South Africa; 3 Biostatistics Unit, South African Medical Research Council, P.O. Box 19070, Cape Town 7505, South Africa; 4 School of Public Health and Family Medicine, Falmouth Building, Faculty of Health Sciences, University of Cape Town, Cape Town 7925, South Africa

**Keywords:** Alcohol, South Africa, Workplace, Safety-sensitive, Employees

## Abstract

**Introduction:**

Harmful alcohol use can compromise worker health and productivity. Persons employed in safety-sensitive occupations are particularly vulnerable to hazardous alcohol use and its associated risks. This study describes the patterns of harmful alcohol use, related HIV risks and risk factors for the harmful use of alcohol among a sample of employees in South Africa working in the safety and security sector.

**Methods:**

A cross-sectional study that formed the baseline for a clustered randomized control trial was undertaken in 2011. A random sample of 325 employees employed within a safety and security sector of a local municipality in the Western Cape Province of South Africa participated in the study. Data were collected by means of an 18-page self-administered structured questionnaire and analyzed using SAS/STAT software version 9.2. For all significance testing, the F-statistic and p-values are reported.

**Results:**

Three hundred and twenty-five employees were surveyed. Findings suggest that more than half (76.1%) of the 78.9% of participants who consumed alcohol engaged in binge drinking, with close to a quarter reporting a CAGE score greater than the cut-off of 2, indicating potentially hazardous drinking patterns. The study further found that employees who use alcohol are more likely to engage in risky sexual practices when under the influence. A favorable drinking climate (p < 0.001) and poor levels of group cohesion (p = 0.009) were significantly correlated to binge drinking.

**Conclusion:**

This study identifies alcohol-related behaviors and associated risks in the context of safety-sensitive occupations at the workplace. It suggests that persons employed within such positions are at high risk for developing alcohol-related disorders and for contracting HIV. This study highlights the need for testing a comprehensive package of services designed to prevent hazardous alcohol use among safety and security employees.

## Background

A healthy and productive workforce is important for the economic success of an organization and also for population health [1,2]. In South Africa, it is estimated that at least 70% of employees engaged in risky alcohol or drug use are in the active workforce, with employers on average losing 86 working days a year due to such absences [3]. The prevalence of risky drinking differs between sectors, with rates as high as 25% reported in the mining industry [4,5]. High levels of risky alcohol consumption are also seen in other sectors. For example, findings from a 1993 study among farm workers in the deciduous fruit industry in the Western Cape Province indicated average usual weekend (Friday–Sunday night) consumption in grams of pure alcohol to be equivalent to the consumption of six 750-ml bottles of wine or a 750-ml bottle of spirits [6]. Similarly, in a more recent study of police officers in the Limpopo province, 55% of officers admitted to risky levels of alcohol consumption [7].

The fact that the problematic use of alcohol is high among persons employed within both the formal and informal working sectors of South Africa is worrying, as alcohol is a major risk factor for communicable and non-communicable diseases, injuries and mental disorders [8]. Additionally use of alcohol at work or just before work can cause serious accidents and work lapses, interfering with work efficiency and precision, further compromising productivity [9].

Running parallel to the high levels of harmful alcohol use in South Africa is the HIV/AIDS epidemic. Sub-Saharan Africa (SSA) remains the region most heavily affected by HIV and AIDS, with negative implications for the organization and the economy [10]. Quantitative studies estimating the cost of HIV/AIDS in 14 large southern African companies, found that HIV/AIDS on average increased employment costs annually by at least 3% [11]. Although the causal relationship between alcohol use and the acquisition of HIV is still inconclusive at this time [12], there are sufficient studies to suggest a link. For instance, a recent meta-analysis of 12 studies found that an increase in blood alcohol concentration of 0.1 mg/ml was associated with an increase of 2.9% (95% CI: 2.0-3.9%) in the likelihood of engaging in unprotected sex [13]. The implication of this is that workers who are risky users of alcohol, could increase their risk of infecting themselves or others with HIV or re-infecting themselves if they are already HIV positive [14] and that such consequences could not only be detrimental to themselves but also to their employers.

Persons involved in safety-sensitive or physically risky occupations are more likely than those in less hazardous positions to have problems related to alcohol [15-17] and consequently be at risk for contracting viruses such as that of HIV. For example, a study in the USA showed that employees in safety-sensitive occupations were 40% more likely to have problematic alcohol or drug use patterns and at least 60% were more likely to have admitted to using alcohol or drugs at work in the previous year [18]. Literature suggests that employees employed in physically and psychologically hazardous positions are more likely to have problems associated with alcohol or drugs when compared to persons who hold jobs that are less risky [17]. Internationally, problematic alcohol and drug usage among employees in this group has driven proposals to address harmful alcohol and drug-related behaviors (and associated risks) through testing initiatives, treatment and early intervention efforts, return-to-work initiatives, peer support programs, and prevention programs to support employees in such positions [19].

Despite high rates of reported harmful alcohol practices among the general population in South Africa and growing rates of HIV infection [2], little is known about the patterns of harmful alcohol use and associated risks such as HIV among persons employed within safety-sensitive positions in this country. This lack of knowledge potentially hampers the development of interventions that address problematic use and related risks in this sub-population. This paper aims to redress this gap by describing the patterns of alcohol use, related HIV risks and risk factors for the harmful use of alcohol among a sample of employees in South Africa working in two divisions of the safety and security sector.

## Methods

A cross-sectional study that formed the baseline for a clustered randomized control trial, was undertaken in 2011–2012 to examine harmful alcohol use, associated HIV risks, as well as risk factors for the harmful use of alcohol among a sample of 325 employees employed within the safety and security sector of a local municipality in the Western Cape province of South Africa. The participating municipality was self-identified by a contact person employed within the Employee Assistance Program (EAP) division of the said municipality. The contact person acted as a broker, facilitating entry into the safety and security department. Employees (within their existing workgroups) were eligible for participation. Intact workgroups were therefore randomly selected to complete a pen and paper self-report questionnaire and to attend an intervention as part of a trial, which is described at length elsewhere [20]. For the purposes of anonymity, the participating municipality will not be named. However to provide a backdrop to the study, it is important to note that two divisions within the municipality’s safety and security department participated in the study. One of the divisions generally respond to emergency and rescue situations and primarily protect society from all types of accidents and emergencies, whilst the second division work in partnership with communities to uphold law and order, ensure public safety and reduce crime. Employees in both divisions are permanent employees of the municipality.

Since the data presented is the baseline findings of a clustered RCT, the sample size calculation was based on the power as calculated for cluster randomized control trials. Calculations depend on two sample sizes (groups and individuals within groups), the intra-class correlation (ICC), and the effect size [21,22]. We anticipated a sample of 40 workgroups (with approximately 10 employees in each) for the field trial. Of these, 20 were randomized to receive the proposed program (intervention arm), and 20 continued with standard procedures (control arm) and received a one hour discussion on wellness. We estimated the ICCs to be .03, such that 3% of total variability in outcomes reflected workgroup differences. Based on this assumption, one hundred and ninety employees were needed in each cohort (n = 380). Although there were no refusals to participate, due to circumstance beyond the control of the researchers, only 325 employees participated.

Written informed consent to participate in the study was obtained from all participants. The consent forms were translated into the vernacular. Confidentiality of employee personal details was ensured by the issuing of unique identifying numbers. Ethical approval for this study was granted by the University of Cape Town’s Health Research Committee.

### Measures

Data were collected by means of an 18-page self-administered structured Workplace Questionnaire (WQ) which included questions relating to sample demographics as well as questions on AOD use, related HIV risk behaviors and risk factors for the harmful use of alcohol.

#### Demographic Items

The self-completed questionnaire included a section providing information on employee age, marital status and their gender. Other information such as length of employment and education level was also included.

#### Alcohol consumption measures

Several items examined alcohol consumption. These items were drawn from the questions developed by the Texas Christian University (TCU) as part of the TCU Workplace Project [17], as well items from the South African Community Epidemiology Network on Drug Use (SACENDU) data collection tool [23]. Questions elicited responses on past 30 day use of alcohol, days in past 30 days a participant had more than five drinks at one sitting (referred to as ‘binge drinking) and whether participants, in the last six months, went to work with a hangover or called in sick because of a hangover. An additional question asked participants whether they thought they had a problem with alcohol. The CAGE questionnaire, a self-report four-item test with questions (on Cutting down, Annoyance at criticism, Guilty feelings and use of an Eye-opener) was also used to screen for hazardous alcohol use. On the CAGE, two or more positive replies suggest hazardous use of alcohol [24].

#### Alcohol-related HIV risk

Seven alcohol or drug-related HIV risk questions were taken out of a 25 item questionnaire developed by Rawson and colleagues focusing on sexual thoughts, feelings and behaviors that patients recollected from the last time they were under the influence of a single psychoactive agent [25]. Participants were asked to give a 0 = ‘No’ or 1 = ‘Yes’ response to the seven questions.

#### Risk factors for the harmful use of alcohol

Scales used to measure risk factors for the harmful use of alcohol were developed by the Texas Christian University (TCU), Fort Worth, Texas [17]. These measures were selected for the following reasons: to measure employee and workplace characteristics at baseline but also at post-intervention and to directly link these changes in dependent measures to participation in the experimental intervention, which has been described elsewhere [20]. In this study scale reliability was tested using Cronbach alpha and corresponding values are reported on for each scale.

#### Individual stress

Variables for individual stress were measured on a scale 1 = ‘strongly disagree’ to 5 = ‘strongly agree’. The 5 items asked employees questions on different dimensions of stress. The Cronbach’s alpha statistic for this scale as measured among the safety and security employees was α = 0.85. In this study the average of items was used as a composite measure of the experience of individual stress.

#### Group stress

The variable group stress assessed several dimensions of stress experienced in the group. Group stress was measured on a 5-item scale, ranging from 1 = ‘strongly disagree’ to 5 = ‘strongly agree’. The Cronbach’s alpha statistic for this scale among study participants was equal to 0.80. The average of items was used as a composite measure of the experience of group stress.

#### Job satisfaction

Employee job satisfaction was measured using a 6-item scale, with 1 = ‘highly dissatisfied’ and 6 = ‘highly satisfied’. Reliability tests attained a Cronbach’s alpha score of 0.85. The average of the items was used a composite measure for job satisfaction.

#### Perceived risk at work

The scale ‘perceived risk at work’ is a 5-item scale measuring degree of risk that leads to lost productivity and/or safety problems. Items were measured on the following 1 = ‘no risk’ to 5 = ‘great risk’. Reliability statistics confirmed a Cronbach alpha of (α = 0.83). The 5 items were averaged to represent an estimate of employee’s perceptions on perceived risk at work.

#### A climate favorable to drinking

Drinking climate was assessed by the frequency of four co-worker behaviors: i) drinking together off the job, ii) talking at work about drinking, iii) getting together just to get drunk, and iv) is alcohol available at work-related parties. Responses ranged from 1 = ‘never’ to 5 = ‘almost always’. Reliability, as measured by Cronbach’s alpha was 0.74.

#### Group cohesion

A 5-item measure of group cohesion was used. Employees rated each item along the same 5-point scale 1 = ‘strongly disagree’ to 5 = ‘strongly agree’. The average of the items was used as a composite measure of perceived group cohesiveness, which had good internal consistency as measured with Cronbach’s Alpha (α = 0.71).

### Analysis

Descriptive analyses (means *μ*; standard deviations and frequencies) were used to describe demographic data; harmful alcohol use and alcohol-related HIV risk variables. Days having any use of alcohol and binge drinking (defined as days having more than five drinks in one sitting in the past 30 days) as well as total scores on the CAGE questionnaire were analyzed as a count variable with a Poisson distribution. A Gaussian distribution was assumed for other continuous variables like age, while a binary distribution was used for the variables with a binary outcome. To assess the degree to which CAGE positivity (scores equal or greater than 2 out of 4) and participant self-report on whether they thought they had an alcohol-related problem agreed, a Kappa statistic was estimated. To examine whether age, length of employment and binge drinking were associated with a CAGE score ≥2, binary multiple regression analysis was used.

To test whether individual stress, group stress, high job stress, a climate favorable to drinking, job satisfaction and levels of group cohesion are associated to binge drinking, multivariate analysis (Poisson regression) was used. The model was therefore built solely on the above variables, supplemented by the inclusion of age and division. All regression models used were adjusted for clustering (considering that questionnaires and the accompanying intervention were administered in employee workgroups), using Proc Glimmix in SAS with cluster as a random effect. For all significance testing, the F-statistic and p-values (< 0.05 for statistical significance) are reported.

In instances where high levels of non-response was recorded, particularly on the use of alcohol, only available data were analyzed. Non-responders were not classified as drinkers or non-drinkers.

## Results

### Demographic information

Three hundred and twenty-five employees were surveyed (Table 1). The mean age of participants was 36.3 years (SD = 6.60). A large proportion of participants were married (62.8%). Eighty seven percent of participants were male and 12.9% female, with the majority (72.3%) having matric (Grade 12) as their highest level of education. Another 17.5% of the participants had some form of tertiary level education. In respect of length of employment, the most common category of work duration was 5–10 year service history (47.2%).

**Table 1:** Demographic information

	***n***	**%**
**Gender**		
Male	281	87.0
Female	42	13.0
**TOTAL**	323*	100
**Age in years**		
20-30 Years	55	17.0
31-40 Years	197	60.8
41-50 Years	61	18.8
51-60 Years	11	3.4
**TOTAL**	324*	100
**Language**		
Afrikaans	134	41.2
Bilingual	1	0.3
English and Afrikaans	14	4.3
English	98	30.2
Sotho	4	1.2
Unknown	4	1.2
Xhosa	69	21.2
Zulu/Swazi	1	0.3
**TOTAL**	325	100
Education*		
GR 7	1	0.3
GR 9	2	0.6
GR 10	14	4.4
GR 11	7	2.2
GR 12	235	74.4
Tertiary	57	18.0
**TOTAL**	316*	100
Marital status*		
Single	72	22.5
Married	214	66.9
Divorced	32	10.0
Widowed	2	0.6
	320*	100
Length of employment*		
6 months to 1 yr	1	0.3
1-5 years	46	14.4
5-10 years	151	47.2
10-15 years	64	20.0
More than 15 years	58	18.1
**TOTAL**	320*	100

*Gender missing cases 2; Age in years missing cases 1; Education missing cases 16; marital status missing cases 2; Length of employment missing cases 5.

### Patterns of alcohol use

Of the 325 employee participants surveyed, 21.3% did not answer the question about alcohol use. Of the 256 participants who did answer, 21.1% reported never using alcohol, while 78.9% reported some use of alcohol during the past 30 days. Of those participants who reported using alcohol, 18.0% indicated that they used alcohol less than monthly, 23.4% reported monthly use of alcohol and a higher percentage (34.0%) reported using alcohol on a weekly basis. A small percentage of employees indicated daily or almost daily use of alcohol (3.5%). When computed among drinkers, the average number of days spent drinking (any use) in past 30 days was reported as 5.6 days (± 5.3). The average number of days on which employees engaged in binge drinking was recorded as 4.4 (±5.4). Seventy-six per cent of employees engaged in binge drinking in the past 30 days.

### CAGE screening test for problematic alcohol use

Table 2 depicts the CAGE cut-off scores. Of those employees who use alcohol, 19.6% reported a CAGE score of ≥ 2, indicating symptoms of alcohol problems.

**Table 2:** CAGE scores

**CAGE SCORE**	**n**	**%**
0	139	64.7
1	34	15.8
2	21	9.8
3	14	6.5
4	7	3.3
Total	215	100

The employees were also asked whether they thought they had a drinking problem; 6.5% reported “yes”, and a further 6.5% indicated that they “may” have a problem with alcohol. Of employees who reported not having an alcohol problem, 87% scored less than 2 on the CAGE; conversely, of workers who reported that they may have a problem, 68% scored 2 or more on the CAGE. Agreement between self-perception of whether they had a drinking problem and cut off on the CAGE questionnaire, estimated as a kappa statistic, was 0.462; 95% CI = 0.3029 -0.602 (Table 3).

**Table 3:** Level of agreement between CAGE scores and employee self-disclosure

**CAGE scores**	**Employee versions of whether they thought they have a problem or not**				**Total**
	**‘I don’t think I have a problem’**		**‘I think I may have a problem’**		
	**n**	**%**	**n**	**%**	
<2 cage score	151	87.28	9	32.14	160
>2 cage score	22	12.72	19	67.86	41
TOTAL	173	100	28	100	201

### Problematic alcohol use, by gender

Mean days of binge drinking was significantly higher for males, μ males = 4.6 (SD = 5.40); μ females = 2.5 (SD = 5.60); F (1,208) = 32.15; p < 0.0001. Males on average drink more than their female counterparts. Although not reaching the set level of statistical significance (p = 0.05), men are more likely to have a CAGE score of 2 and more when compared to women, F (1,209) = 2.96; p = 0.08.

### The association of age, length of employment and binge drinking with a greater than 2 CAGE score

The results suggest that the number of days on which participants engaged in binge drinking, was significantly associated with a high CAGE score (Table 4). The significant and strength of this association was unchanged when controlled for age and length of employment, neither of which were independently associated with a high CAGE score, both as individual models and when combined with binge drinking in one model (t = 0.56; p = 0.57 and t = 0.49; p = 0.62 respectively).

**Table 4:** Binary multiple regression model: association between days having >5 drinks, age and length of employment to a >2 CAGE score

**Independent variables**	**Model 1**^**1**^				**Model 2**^**2**^				**Model 3**^**3**^				**Model 4**^**4**^			
	**Estimate**	**SE**	**t-value**	**p- value**	**Estimate**	**SE**	**t- value**	**p- value**	**Estimate**	**SE**	**t- value**	**p- value**	**Estimate**	**SE**	**t- value**	**p- value**
Days having more than 5 drinks	0.134	0.029	20.14	<0.001									0.133	0.030	4.41	<0.001
Age					0.014	0.025	0.56	0.575					0.011	0.034	0.32	0.752
Length of employment (0–15 years)									0.176	0.358	0.49	0.624	0.152	0.431	0.35	0.726

^1^Model 1: the association between days having more than 5 drinks and a >2 CAGE score.

^2^Model 2: the association between age and a >2 CAGE score.

^3^Model 3: the association between length of employment and a >2 CAGE score.

^4^Model 4: entered into the model were all three independent variables (more than five drinks, age, employment), and the association to a >2 CAGE score was explored.

### Drinking alcohol at work

Employees were asked whether in the last six months they had one or more drinks at work. Six per cent of employees indicated that they had one or two drinks in the past 6 months during their lunchtime break. Four per cent of employees reported that they had more than two drinks during their lunch hour at work in the past 6 months.

### Going to work with a hangover or calling in sick because of a hangover

Thirty-one per cent of employees indicated that, in the course of the past 6 months, they had come to work with a hangover. Of the 31 per cent who reported going to work with a hangover, 18.7% reported doing so less than monthly, 8.4% indicated going to work with a hangover on a monthly basis and 3.7% indicated that they did so on a weekly basis. In respect of missing work or calling in sick because of a hangover, 16.7% employee participants reported having called in sick in the last 6 months because of a hangover.

Divisional differences in calling in sick were however noted. Employees from the division that deals mostly with the enforcement of law and order were more likely to call in sick (20.4%) when compared to their colleagues in the other division (4.7%). Adjusting for clustering confirmed a significant difference between the two divisions (F (1,212) = 9.42, p = 0.002).

### Alcohol-related HIV risks

Of those employees who engage in the harmful use of alcohol, 10.8% indicated that their sexual drive increases with the use of their primary substance of abuse. In addition ten percent were likely to have had sex with someone other than their main partner when using substances, and 8.6% indicated having sex with a casual partner when under the influence of alcohol. At least a quarter of employees who completed the survey indicated that they engaged in more than one or multiple sexual risk behaviors (Table 5).

**Table 5:** Degree of sexual risk (n = 325)

**Degrees of risk**	**n**	**%**
0	171	69.5
1	29	11.8
2	14	5.7
3	14	5.7
4	9	3.7
5	5	2.0
6	2	0.8
7	2	0.8
TOTAL	246*	100

*Missing cases (n = 79).

There was a significant difference in the degree of risk for drinkers when compared to non-users. Drinkers were significantly more likely (μ = 1.06; SD = 1.65) to have one or more HIV risk exposure (for example, sex with multiple partners) when compared to their non-using counterparts μ = 0.09; SD = 0.37; F (1,155) = 17.62; p < 0.0001.

### Risk factors for binge drinking: individual stress, group stress, job satisfaction, perceived risk at work, a climate favorable to drinking and levels of group cohesion

Results from multiple regression revealed that age (demographic variable), a favorable drinking climate and poor group cohesion were found to be significant correlates of binge drinking, in a positive direction (Table 6). Job satisfaction and perceived risk at work were also significantly correlated to binge drinking, although this was not in the anticipated direction.

**Table 6:** Poisson regression depicting the relationship of psycho-social and drinking climate variables to binge drinking

**Independent variables**	**Estimate**	**SE**	**t-value**	**p-value**
Division	0.099	0.253	0.39	0.698
Age	0.012	0.006	2.00	*0.047*
Individual stress	0.050	0.050	1.00	0.320
Group stress	−0.031	0.062	−0.49	0.622
Group cohesion	−0.164	0.062	−2.65	*0.009*
Drinking climate	0.187	0.045	4.19	*<0.001*
Job satisfaction	0.216	0.049	4.44	*<0.001*
Perceived risk at work	−0.157	0.042	−3.73	*<0.001*

## Discussion

The study findings suggest a serious challenge posed by risky alcohol use in this population. Of the sample surveyed, sixty-two percent indicated past 30 day use of alcohol. This percentage is much higher than the provincial prevalence reported for adults (44.8% for both sexes) in the South African National HIV Incidence, Behavior and Communication Survey (SABSSM) of 2008 [26]. Additionally, findings from our study suggest that more than half of the participants who reported consuming alcohol engaged in binge drinking (76.1%), a figure far higher than equivalent provincial estimates of past month binge drinking of 16.3% in the SABSSM survey [26].

In attempting to understand problematic use of alcohol evident in this population, it is important to consider whether the occupations of the sample studied potentially place them at more risk for developing alcohol-related problems. It has been reported elsewhere that safety-related occupations are associated with higher alcohol or drug use during or before work when compared to other non-safety related occupations [7,27-29]. This suggests that employees employed in physically and psychologically hazardous positions are more likely to have problems associated with alcohol or drugs when compared to persons who hold jobs that are less risky [17]. This may explain the higher rates of usage in this sample when compared to population estimates such as the SABSSM study, since this is a selected high-risk population. This points to the importance of regulations prohibiting the performance of safety-sensitive functions when employees are considered to be under the influence, and further highlights the importance of continued prevention and early intervention initiatives as strategies for helping both employers and employees [30].

In trying to further unpack the extent of problematic alcohol use, the study used the total CAGE score. Our findings suggest that of those employees who indicated use of alcohol, close to a quarter had a CAGE score greater than the cut-off of 2, suggesting potentially hazardous drinking patterns. Agreement between self-reported alcohol problems and a CAGE score, as measured in a Kappa statistic was moderate (kappa = 0.46). This is consistent with other findings that very often persons exhibiting problematic use of alcohol do not acknowledge their behavior as problematic [31], and may go about their daily work when they are in actual fact impaired. This places the affected employee and their co-workers at risk. It also highlights difficulties in relying on self-report measures in alcohol related studies, particularly in identifying risky drinking, where validated and reliable measures should be preferably used. This finding is also significant since it highlights a need for early interventions and early screening to form part of workplace-based interventions to increase awareness of problematic alcohol use. A failure to routinely screen employees for possible harmful use of alcohol may have significant impacts on work performance, which would include job-related injuries, job withdrawal and even certain antagonistic work behaviors [9,32].

The study further found that males were significantly more likely than females to have a ≥2CAGE score or engage in binge drinking. Various studies have alluded to working men being more at risk for alcohol use disorders when compared to their female counterparts [33,34]. This is altogether not surprising considering that multiple data sources in South Africa [35-37] all find that men are more likely to seek treatment for alcohol and drug-related problems. The study also found no significant association between a positive score on the CAGE and age or length of employment, which suggests that age and length of employment do not predict a positive CAGE score. In a study of police officers researchers found that cirrhosis of the liver was elevated across all years of services [38]. The development of a substance abuse problem is not dependent on age or length of employment but a combination of environmental, biological and other psycho-social aspects [33]. This finding highlights the importance of aiming interventions at employees independent of their age and length of service.

In relation to drinking at work almost 10% of employees admitted to using alcohol during their lunch break at work. In a large scale survey of persons employed with the railway services in the USA, a significant proportion (ranging from 6%-24%) of respondents reported drinking at least once whilst at work [39]. In a separate study of military personnel close to 10.1% reported drinking just before or during work [40]. This suggests that use of alcohol during work is not unique to this study but appears to be a global problem in stressful workplaces. This is worrying as it significantly increases the risk of work-related injuries and poor judgment especially in the context of this sample who are engaged in safety-sensitive jobs [17]. Drinking at work highlights a serious disregard for policies regulating the use of alcohol and drugs within the workplace. Typically employees within an organization should be aware of rules stipulated in the guidelines and the sanctions involved [41]. As part of organization preventative measures, screening and awareness of the in-house alcohol and drug policy should become part and partial of staff inductions, policy training and overall wellness.

This discussion is also relevant to the finding that a third of employees indicated going to work with a hangover. Employees engaging in drinking when away from their work are very likely to experience a variety of adverse consequences and problems such as hangovers which affects performance and employee cognitive and motor functions which are risks for bad judgment and other interpersonal conflicts and injuries [9,33,39]. For example, a study of hangover effects on pilots found that pilots in a flight simulator who had a blood alcohol count of 0.10 per 100 ml blood the night before, had significantly poorer responses on handling the aircraft [39]. Working in a hangover state slows reaction time, impairs the lateral field of vision severely, and reduces cognitive processing of information and eye-hand coordination [33]. Additionally, work-related drinking and hangovers also impact on worker productivity and quality of working life, for instance, workers are more likely to get into disputes at work or with family [39]. Drinking also increased the likelihood of calling in sick for work the next day. This finding was particularly significant for employees in the law enforcement division, since these employees (as part of the ‘reporting for duty protocol’) are exposed to early morning parades. A time-series study in Norway found that a one liter increase in the total alcohol consumption in the period 1957–2001 was associated with a 13% increase in sickness absence [42]. Similar studies have also found a relationship between alcohol use and absenteeism [33,43,44].

Compounding the problem is the finding that a third of employees in our sample, who also use alcohol, reported engaging in risky sexual practices when under the influence. Alcohol use prior to sex has been reported to increase the rate of unprotected sex [45-47] which could result in exposure and subsequent contraction of HIV/AIDS and other sexually transmitted infections (or reinfection if a person is already HIV positive). Heavy use of alcohol is associated with disinhibition and impaired judgment that may lead to sexual risk behaviors such as inconsistent condom use or sex with multiple partners [48], as evidenced in this study. Similar results were found in a study of female commercial sex workers living near to single-sex hostels of mineworkers. It was reported that 68.6% of sex workers and 28.6% of mineworkers were HIV positive, with mine workers reporting high levels of unprotected sex (sex without a condom) as well as heavy consumption of alcohol [49]. These associations between substance abuse and HIV risk point to the need for interventions that address problematic alcohol use and related sexual risks simultaneously.

Lastly, literature on substance abuse in the workplace has consistently linked problematic alcohol use to a favorable drinking climate, low group cohesion, group stress and high job risks [50]. For instance, Trice and colleague found that good social (team) support within the work context assists in curbing problems related to risky drinking climates [51,52]. In this study, a climate favorable to drinking and poor group cohesion emerged as a predictor for engaging in binge drinking. Job satisfaction and perceived job risk were, however, negatively correlated. This is surprising since literature suggests that persons in safety-sensitive jobs have more problems associated with alcohol or drugs when compared to persons who hold jobs that are less risky [17]. In relation to perceived job risk, a possible explanation may be that those who realize the implications of drinking at work are less likely to binge drink. This awareness could act as a restraint on possible harmful behavior. Additionally, the results could be influenced by low response to these variables or can imply that at the time of this study, there may have been other factors that represent stronger predictors of binge drinking. Regardless, structures such Employee Assistance Programs or other psychological services should be called upon to provide safety and security employees with programs that increase employee hardiness through coping enhancement programs, supervisor support, and group cohesion [53].

While our findings provide insights into the extent of problematic alcohol use among safety and security employees at a local municipality, they should be interpreted in the context of certain limitations. Firstly, the sample used in this study was drawn from one municipality in the Western Cape Province and may not be representative of or generalizable to all employees within safety and security occupations. Furthermore, alcohol use was measured using single item measures and the CAGE instrument. The study would have benefitted from the use of other standardized instruments that measure severity of alcohol use by providing cut-off scores for hazardous or harmful use. Nor was the measure used to collect responses on alcohol-related HIV an established, reliable, or validated instrument, though it has been used in at least one other context [25]. Use of a validated alcohol-related HIV risk scale for examining such risks is therefore recommended. Additionally, the study used a Kappa statistic to determine level of agreement between the CAGE and employee self-report on problematic alcohol use. It should be noted that the CAGE screening tool, although a validated and reliable instrument, is not in any way the gold standard and self-report measures were used as a proxy for calculating level of agreement since the potential for over or under-reporting is high for both.

There were problems with non-response and missing data. A major limitation was the high number of employees not answering the question about use of alcohol (21.3%), weakening the power of the analyses performed. Recent literature cautions researchers against assuming reasons for dropout or in the case of this study non-response [54,55] with no supporting information, since this introduces bias. Since reasons for non-response were not explored, the findings could probably reflect an under- or overestimate of the true extent of problem drinking in employees from the two sectors studied. Additionally participant responses to the alcohol-related HIV risk questions were also poor. This section of the study was also burdened by the problem of under- and over-reporting, since participants either fabricated information on, for instance, how many times they had sex in the past month (using inflated figures such as 2000 times) or indicated quite clearly that the information was none of the researcher’s business.

In relation to future research, studies examining alcohol and other drug use in a cross-section of industries (including other safety and security occupations) are needed, since this will facilitate a better understanding of the nature and extent of alcohol and drug use in South African workplaces. This should ideally be followed with testing the efficacy of broad-based prevention programs in the workplace ensuring that such programs are culturally appropriate and fit the South African context. These should be part of an effort to reduce both problematic alcohol and drug use, but should also recognize and seek to address the problem of alcohol-related HIV.

Although one of the objectives in this study was to explore both alcohol and drug abuse, underreporting on drug abuse resulted in an inability to undertake any useful bivariate or multivariate analysis. Further studies should aim to examine both alcohol and drug abuse and address possible underreporting with the inclusion of biological markers, which will provide more information on the extent of drug abuse. Additionally, further studies should examine factors that act as a protection from problematic alcohol use among persons in safety and security occupations as well as persons in general employment. Lastly and considering the problem of non-response encountered in this study, future research should go to even greater lengths to reassure participants of the confidentiality/anonymity of data collection and how findings will be reported especially in situations when only a small number of participants come from a division or sector.

## Conclusion

Despite its limitations, the findings from this study suggest that persons employed within safety and security positions are potentially at higher risk for developing alcohol-related disorders and are also at risk for contracting HIV and other STDs. This highlights the need for testing a comprehensive package of services that are designed to prevent the harmful use of alcohol among safety and security employees.

## Competing interests

The authors declare that they have no competing interests.

## Authors’ contributions

NHB contributed to the conceptualization and writing of the manuscript and analyzed the data; RL contributed to data analysis; CP and LL contributed to the design of the study, writing and reviewing the manuscript. All authors read and approved the final manuscript.
